# On the Ambient Optic Array: James Gibson’s Insights About the Phenomenon of Chiaroscuro

**DOI:** 10.1177/2041669520952097

**Published:** 2020-09-30

**Authors:** James T. Todd

**Affiliations:** Department of Psychology, Ohio State University

**Keywords:** three-dimensional perception, surfaces/materials, contours/surfaces, shapes/objects

## Abstract

In 1966, James Gibson first presented his theory of the ambient optic array, and he proposed a new field of ecological optics that he hoped would advance our knowledge on this topic. This study will consider how his ideas have largely come to fruition over the past 50 years. It reviews the research on the visual perception of three-dimensional shape from shading, the effects of ambient light from surface interreflections on observers’ perceptions, the perception of the light field, and the perception of surface materials. Finally, it also considers Gibson’s impact on these developments.

During the Italian renaissance, there were two important discoveries that dramatically improved the abilities of artists to create realistic depictions of three-dimensional (3D) scenes. The first of these was linear perspective, which involves patterns of lines that converge toward a vanishing point. The second involves patterns of light and shade (called chiaroscuro). Leonardo da Vinci did extensive observations of these patterns, which he documented meticulously in his notebooks with many illustrations. Within the academic study of visual perception, linear perspective has received an enormous amount of attention and has been the subject of numerous empirical investigations. Chiaroscuro, in contrast, has received almost no attention until quite recently. If it is mentioned at all in most textbooks on visual perception, it is typically listed in passing as one of many possible depth cues, with no discussion about how it influences perception, or why variations of light and shade occur in the natural environment.

The first serious attempt to answer these questions was proposed by James Gibson (1966) in his second book *The Senses Considered as Perceptual Systems*. In Chapter 10 of that book about visual information, Gibson introduced his theory of the ambient optic array in which he described how the pattern of light at a point of observation is structured by surfaces in the surrounding environment. He elaborated this theory over a sequence of stages, each of which added an additional element of complexity. The first stage of his analysis is represented by Figure 1. In this scene, there is a single luminous object but no reflections off any visible surfaces. Gibson argued that light from a luminous source without any surface reflections provides no useful information for a perceptual system.

**Figure 1.**
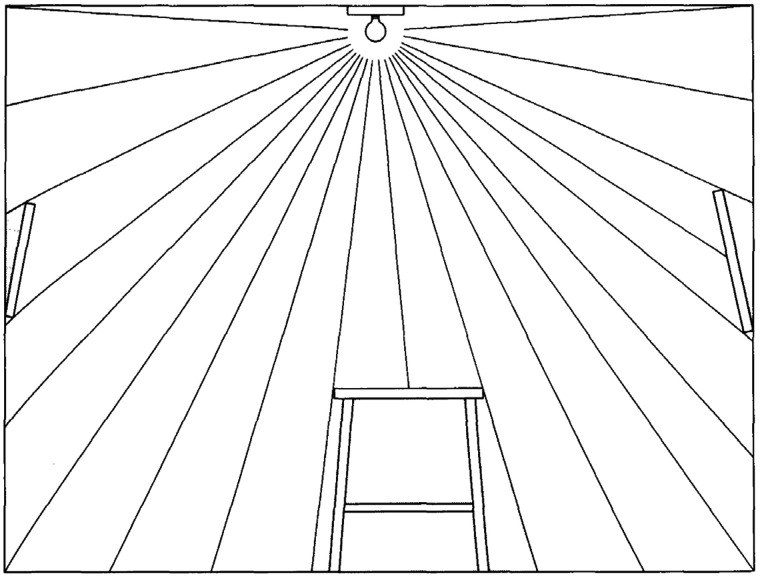
Pure radiant light in a room. Adapted from Gibson (1966).

The second stage of Gibson’s analysis of the optic array is represented in Figure 2. It shows the same scene as in Figure 1 with the addition of surface reflections. It is best to describe this figure in Gibson’s own words. The reflection that is represented in this figure:

**Figure 2.**
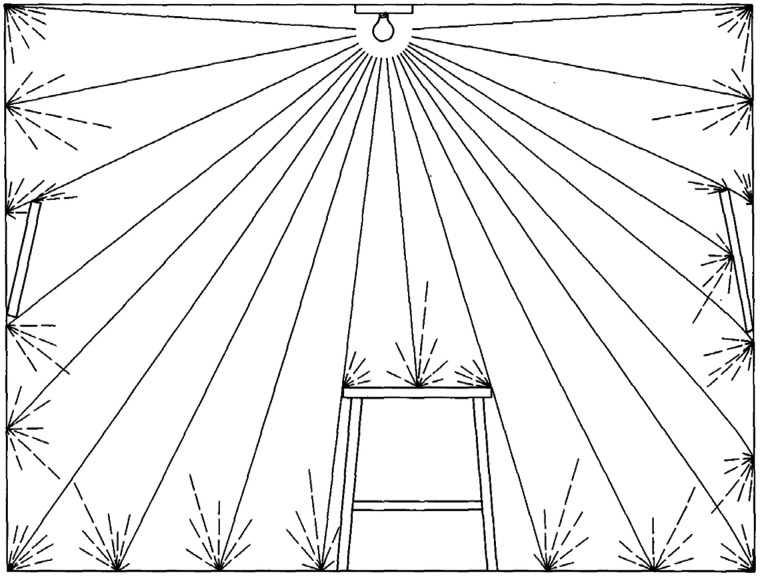
Reflected light from facing surfaces. Adapted from Gibson (1966).

… is not the abstract reflection of a single ray at an ideal mirror, for the surfaces indicated are imperfectly smooth, and a beam is therefore scattered in various directions depending on the microstructure of the surface—that is, on the arrangement of a large number of “micromirrors,” each being a tiny facet of the surface. This scattered or diffuse reflection is characteristic of environmental surfaces. (Gibson, 1966, p. 191)

Gibson also noted how the directional distribution of scattering varies among different surface materials, as shown in Figure 3. For surfaces that appear matte, for example, the scattering is distributed over a broad range of directions, whereas those that appear glossy have distributions that are more tightly clustered within a small range of directions.

**Figure 3.**
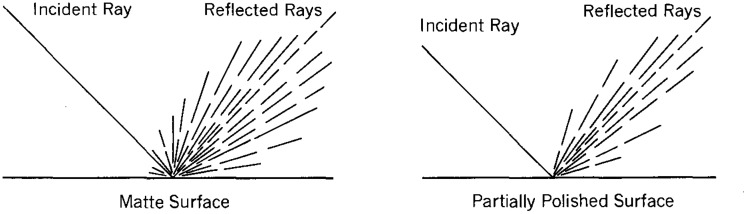
Scattering patterns for matte and partially polished surfaces. Adapted from Gibson (1966).

It is important to emphasize how Gibson’s description of light scattering at a microscopic scale was a radical departure from anything that had been proposed prior to that in the literature on visual perception.

The concept of optical scattering in surface reflection was first introduced by the Swiss physicist Johann Lambert in 1760, but there were few if any perceptual psychologists who had any knowledge of that work in 1966. Because Gibson did not cite Lambert, or any more modern sources on surface scattering, it is reasonable to speculate about how he arrived at his own ideas on this topic. I suspect he may have been influenced by his contacts in the field of illumination engineering, where knowledge about the physics of reflection would have been much more sophisticated than what he could have learned from the literatures on physical optics or visual perception during that period.

It should be noted in Figure 2 that only the direct reflections from the luminous source are included. In Stage 3 of his analysis, he added the additional complexity of surface interreflections, but he was unable to devise a figure to visualize that. He described it as follows:

The result of scattering when there is a layout of facing surfaces is multiple reflection or reverberation. The light bounces from surface to surface endlessly. At this stage the environment is said to be illuminated. An infinitely dense network of rays is formed, consisting of pencils of rays intersecting at every point in the medium. This network of convergence and divergence cannot be represented and must be imagined by the reader. It ceases to exist the instant the source is extinguished. (Gibson, 1966, p. 191)

It should again be emphasized that Gibson’s description of the space-filling pattern of intersecting rays was a radical departure from any previous discussion about the behavior of light in the literature on visual perception at that time. However, there were some closely related ideas that had been proposed in the field of illumination engineering, as I shall describe later.

Stage 4 of Gibson’s analysis focuses on the pattern of converging rays at a single point in space. Stage 5 considers the sampling of optical structure at that point by a stationary observer, and Stage 6 discusses how the observer can obtain additional information by moving through space to sample the converging rays at multiple locations. This last stage is represented in Figure 4.

**Figure 4.**
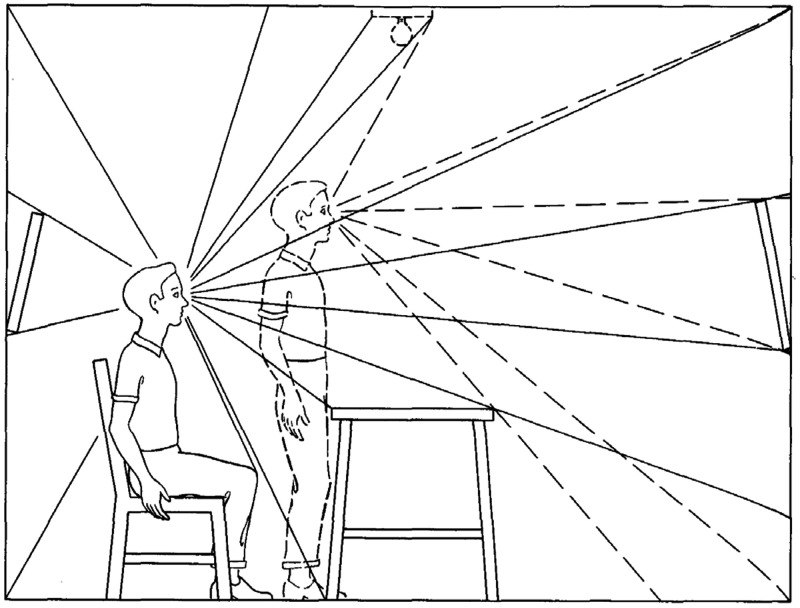
Sampling the convergent rays at multiple vantage points. Adapted from Gibson (1966).

Later on in this chapter, Gibson discusses the causes of structure in reflected ambient light, and he provides a clear explanation of why chiaroscuro occurs:

A facet of the world that faces the sun will be strongly illuminated and will reflect a considerable amount of energy. A facet that is inclined to the sun’s rays, or parallel to them, will not be strongly illuminated and will not reflect much energy. It is said to have less “brightness.” A facet that faces away from the sun’s rays is weakly illuminated, reflects little energy, and is said to have an “attached shadow.” (Gibson, 1966, p. 208)

He then follows this description with an aerial photograph of a hilly terrain like the one shown in Figure 5 to demonstrate how variations in the facing angle of a surface results in systematic gradients of intensity in the reflected light.

**Figure 5.**
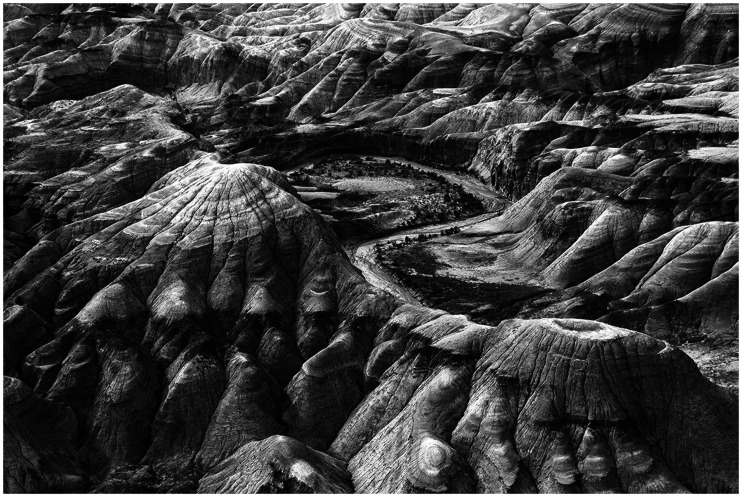
An aerial photo of a hilly terrain. The variations of surface orientation produce gradients of intensity in the pattern of luminance.

Although Gibson’s (1966) book was widely acclaimed, the depth of his insight about the ambient optic array was never fully appreciated, even among his followers. He was just too far ahead of his time. I remember being told by my professor in an undergraduate perception class that the optic array is no more than a renaming of the retinal image. Although I did not object at the time, it is clear in retrospect that this comment was ridiculous. There is nothing in the concept of the retinal image that would naturally lead to the idea that reflected light is scattered in many directions based on microscopic surface structure that is not visible to the naked eye, or that this scattered light would reverberate through space to form a dense network of intersecting rays at every location, or that variations in the local orientation of a surface (i.e., curvature) produce gradients of intensity that provide visual information about 3D shape. In most textbooks on perception, the structure of retinal images is *explained* by projective geometry. However, this does not account for chiaroscuro. Why are there systematic variations of shading across different locations of an image? Projective geometry has nothing to offer concerning that phenomenon, whereas Gibson provides a potential explanation that is firmly grounded in ecological optics.

Gibson was well aware that his proposals about the optic array were a work in progress, as is demonstrated by the following passage:

Anyone who has studied modern optics as this mixed body of knowledge exists today will be puzzled and perhaps offended, by what is here called ecological optics. Such a discipline does not, in truth, exist. But it purports to be a new basis for a science of vision, put together from parts of physical optics, illumination engineering, ecology, and perspective geometry. It is part of the author’s attempt to reformulate more precisely the concept of the stimulus in psychology. (Gibson, 1966, p. 221)

In the remaining portions of this article, I will try to document how far we have come over the past 50 years toward achieving the goal of ecological optics that Gibson so clearly articulated. It is not at all surprising that he did not list computer graphics as one of the fields that would contribute to this enterprise because computer graphics barely existed in the 1960s. However, as I will demonstrate in the discussion that follows, much of the progress in this area is due directly or indirectly to the practical need of producing computer generated images of surfaces that are accurate simulations of the natural environment.

## The Emerging Investigation of Chiaroscuro

The first commercially available computer graphics systems began to appear in the 1960s. They all employed cathode ray tubes, whose front faces were coated with phosphors that glow when hit by an electron beam. If you wanted to draw a square on the screen, you had to program the beam to move in a square path. These systems did not typically allow the intensity of different regions to be manipulated beyond on and off.

During the 1970s, a new technology emerged that wedded traditional computer graphics with television. In these systems, the electron beam always moved in a fixed sequence of horizontal scan lines called rasters, and the intensity of the beam at each pixel location was determined using a large array of memory called a frame buffer. Raster graphics made it possible for the first time to manipulate the gray scale values in an image, and it launched an intensive effort to create images that appear so realistic they are indistinguishable from photographs (i.e., photorealism). This led researchers in computer graphics to consider many of the things discussed by Gibson such as how illumination is scattered by surfaces, and how it is structured by the pattern of surface interreflections. It also made it much easier for researchers in human vision to psychophysically investigate the perception of shape and material properties from shading.

My own involvement in that research occurred almost entirely by accident. As a postdoc at the University of Connecticut in the late 1970s, I was in charge of the psychology computer laboratory. On the advice of a colleague in computer science, I decided to purchase a new raster graphics system primarily because it allowed users to create video tapes of motion displays directly from the video output of the monitor. When the salesman asked me how many planes of memory to include, I naively responded that one would be sufficient because memory was expensive, and I did not see any need to have more than two possible gray scale values of on and off. However, when the system finally arrived, it contained eight planes of memory rather than one, which made it possible to produce 256 distinct levels of image intensity. Although I paid little attention to this enhanced capacity, there was a first year graduate student in the program named Ennio Mingolla who decided to play around with it. He drew a circle in the center of the screen with a linear gradient of intensity that decreased radially from the center and surrounded that with a set of random dots. It looked like a spherical planet in space surrounded by distant stars. He then called me into the laboratory to show off his creation and asked why it worked. I had no idea.

**Figure 6.**
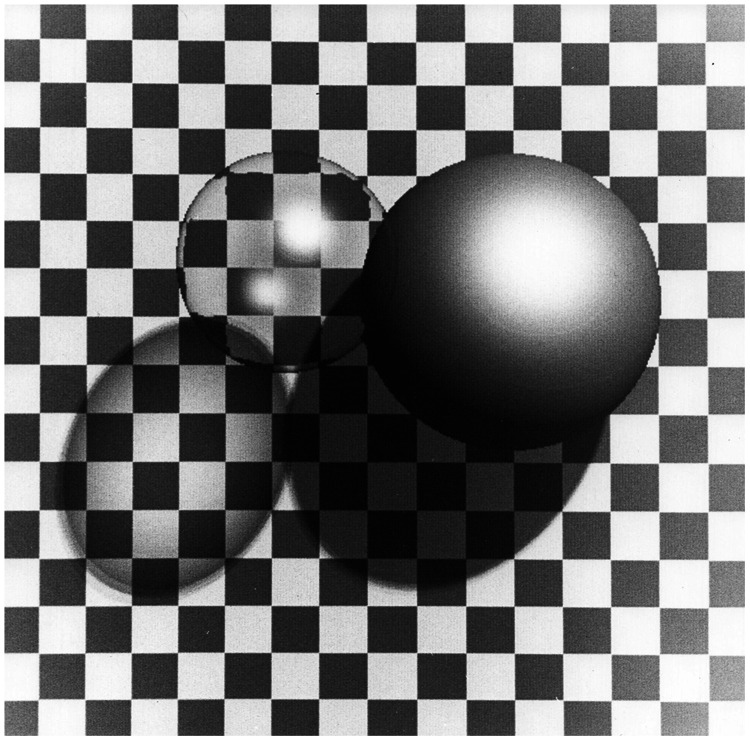
An image of two balls created by Todd & Mingolla (1983).

To rectify that, we went on a crash course in computer graphics to learn about shaders and reflectance models. Ennio eventually did his doctoral thesis on the perception of shape from shading. Our first paper on this topic (Todd & Mingolla, 1983) included a tutorial on shaded graphics in an effort to inspire other researchers in the field. It also included the image shown in Figure 6 to show off some of the things that were possible with this new technology. Ennio first presented this image at a talk he gave at a meeting of the Psychonomic Society in 1981. During his talk, I was seated next to Jacob Beck and Jackie Gibson. When this slide appeared, they both did a double-take. Jackie leaned over to me and asked incredulously *Is that from a computer?* Although this image looks rather primitive by today’s standards, it was much more impressive 40 years ago.

It was not long before other laboratories started to exploit this new technology to study the perception of shape from shading. Heinrich Bülthoff began a similar research program when he was a postdoc at Massachusetts Institute of Technology (MIT) in the 1980s (Bülthoff & Mallot, 1988), and Ramachandran (1988) began to investigate this issue at about the same time.

During this same time period, other researchers in computer vision were starting to develop computational algorithms that could determine the 3D shape of an object from the pattern of shading within a visual image. This all began with the pioneering work of Bertholdt Horn (1975) at MIT, but it quickly became a cottage industry within the field of computer vision (e.g., see Ikeuchi & Horn, 1981; Koenderink & van Doorn, 1980; Lee & Rosenfeld, 1985; Pentland, 1984).

Almost all of the research on shape from shading in psychology and computer vision described thus far involved very limited conditions similar to Gibson’s stage 2. Only direct reflections off matte materials were considered, and the lighting was restricted to a collimated beam in a single direction without any cast shadows. With a few salient exceptions, the role of ambient light from surface interreflections did not receive serious attention until the 1990s.

## Let There Be (Ambient) Light

As I mentioned earlier, Gibson was not the first researcher to recognize the complexity of the crisscrossing patterns of rays that fill the volume of space. That honor goes to the Russian mathematical physicist Andrey Gershun. In 1939, he published a monograph entitled *The light field*, which was later translated into English and published in the United States in 1939. The following passage is from the introduction of that paper:

… we shall introduce the concept of the light field, as a part of space studied from the standpoint of transmission of radiant energy within that space. Until recent times, photometry limited itself to concepts concerning the emission and absorption of light by bodies, while the transmitting medium was ignored. The older photometric science was a peculiar manifestation of the concept of actio in distans. The modern study of the light field consists of an investigation of the space-distribution of luminous flux. The separate photons are disregarded and the assumption is made that radiant energy is continuous in time and in space and that the flux of this radiant energy varies continuously from point to point. The familiar method of vector analysis is used in the theoretical study of the light field. (Gershun, 1939, p. 56)

**Figure 7.**
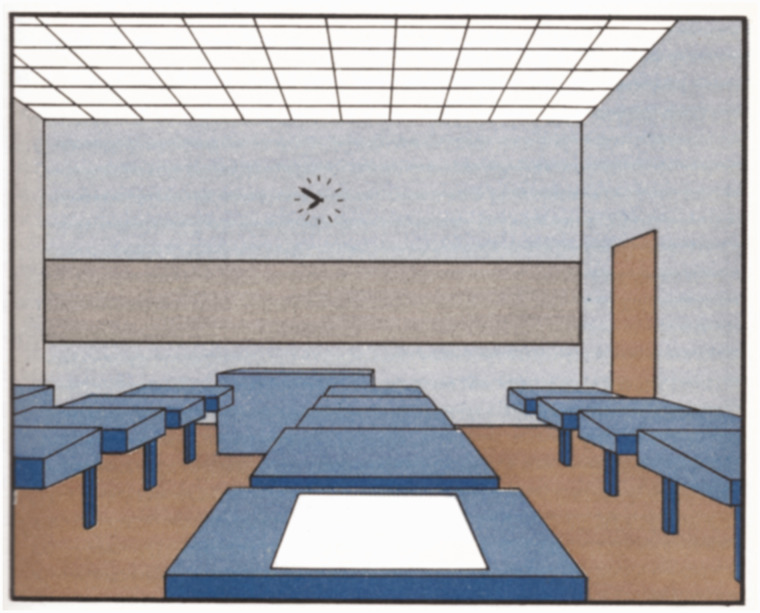
The first computed image that incorporated global illumination. Adapted from Moon & Spencer (1948).

One of the translators of Gershun’s monograph was the mathematical physicist Parry Moon at MIT. He and his wife Domina Spencer have also made numerous contributions to our understanding of lighting in cluttered environments. In 1948, they published a textbook on *Lighting Design* that was used to teach students in architecture at MIT and Tufts University. It included a whole chapter on surface interreflections, and it also discussed how the global effects of multiple reflections can be computed using algorithms developed by Henry Higbie (1934). To help students visualize the accuracy of these computations, they made perspective drawings of rooms and calculated the relative amount of shading for each surface. They then filled in each polygon of their drawings with Munsel papers that matched the results of their calculations, and these were finally ironed together to complete the composite image. An example image from this painstaking process is presented in Figure 7. This image is historically important because it is the very first computed depiction of a scene that includes both direct and indirect reflections—what is now referred to as global illumination. Moon and Spencer (1981) later published a second book on *The Photic Field*, in which they employed a vector field analysis to calculate the lighting in a scene, as first suggested by Gershun.

It is interesting to speculate about how much Gibson may have known about these earlier contributions. It is clear he was familiar with the field of illumination engineering, because that is one of the disciplines he listed that would need to be absorbed into the new field of ecological optics. It is reasonable to assume therefore that he had read some of the content of that field, or perhaps had conversations with some of its practitioners, who would have been familiar with the concepts of surface scattering and indirect reflections. I suspect it is most likely that Gibson’s knowledge of illumination engineering may have strongly influenced his understanding of these topics, even if he was unfamiliar with the most relevant original sources such as Higbie (1934), Gershun (1939) and Moon and Spencer (1948).

With the rapid growth of computer graphics in the 1980s, the computation of global illumination attracted much broader attention than when this problem was confined to illumination engineering. The ray tracing algorithm developed by Whitted (1980) was one of the first attempts to track light rays over multiple bounces, but it was designed primarily for shiny surfaces that produce minimal scattering. Goral et al. (1984) at Cornell University were the first computer graphics researchers to compute global illumination for diffuse reflecting matte surfaces. Their algorithm was adapted from earlier models of heat diffusion, which is closely related to light transport. A similar approach was later employed by Kajiya (1986). He introduced the generalized rendering equation for computing global illumination, and he also developed techniques for approximating a solution using Monte Carlo sampling in order to speed up the required computations. Many other refinements have been added to these methods over the years in an effort to make them faster and to eliminate artifacts in the resulting images.

When these methods were first developed in the 1980s, they required massive amounts of computing time on the fastest computers that were available. Things have improved somewhat today in that the algorithms are more efficient and computers are much faster, but the computation of global illumination is still quite taxing on computer resources. In my laboratory, for example, I have a computer cluster with 96 Xeon core processors. However, even when they are all running in parallel, it can still take several hours to render an image depending upon its size, the composition of the depicted scene, and the level of quality I am trying to achieve.

Given the inaccessibility of these resources within perceptual psychology until quite recently, it is remarkable that the first psychophysical experiment on global illumination was performed in the early 1980s by Gilchrist and Jacobsen (1984). This was only possible because they used real scenes rather than computer simulations. The issue they sought to address was whether observers would exhibit lightness constancy if all the surfaces in the visible environment had exactly the same reflectance. To achieve this, they placed two identical sets of objects inside large wooden boxes that could be illuminated with an unseen light source and viewed through a small peep hole. All of the surfaces in one box were painted black, whereas all of the surfaces in the other were painted white. The results showed clearly that observers could accurately judge the lightness of each scene even when they both had the same mean luminance.

Using a spot photometer to measure the local luminance in different regions, Gilchrist and Jacobsen were able to show that the luminance variance provides the relevant information for distinguishing black and white rooms. In a black room that reflects a small proportion of the incident light the surface interreflections are negligible, and there are large differences in luminance between those regions that receive direct light and those that are in shadows. However, in a white room that reflects a high proportion of the incident light, the light can bounce around off many surfaces before its energy is dissipated. This causes the shadows to become washed out, which reduces the level of contrast in an image.

**Figure 8.**
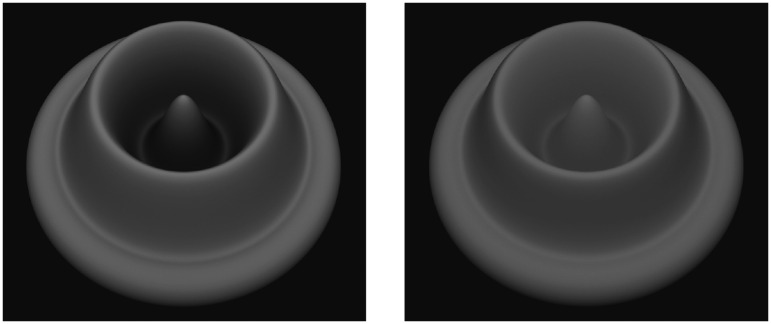
Effects of vignetting and indirect illumination. Both surfaces are illuminated by a diffuse overhead source. The surface on the left has a reflectance of 0.5 and the one on the right has a reflectance of 0.9.

The effects of surface interreflections are greatest in concave surface regions because the light becomes trapped in those regions and illuminates the same surfaces on multiple bounces. This effect is demonstrated in Figure 8, which originally appeared in Todd et al. (2015). Both images in this figure depict a radial cosine surface with diffuse overhead illumination as on a cloudy day. The surface depicted on the left has a reflectance of 0.5. Note that the inner part of the circular ridge appears darker than the outer part. This is because much of the sky is occluded in those regions so that they receive less illumination—an effect that is referred to as vignetting (Koenderink & Van Doorn, 2003; Langer & Zucker, 1994). For the surface on the right, the reflectance has been increased to 0.9, so that the light undergoes a greater number of bounces before all its energy is dissipated. Note in that case that the brightness of the internal part of the ridge has been increased substantially. The brightening of the interreflections effectively outweighs the darkening caused by vignetting.

**Figure 9.**
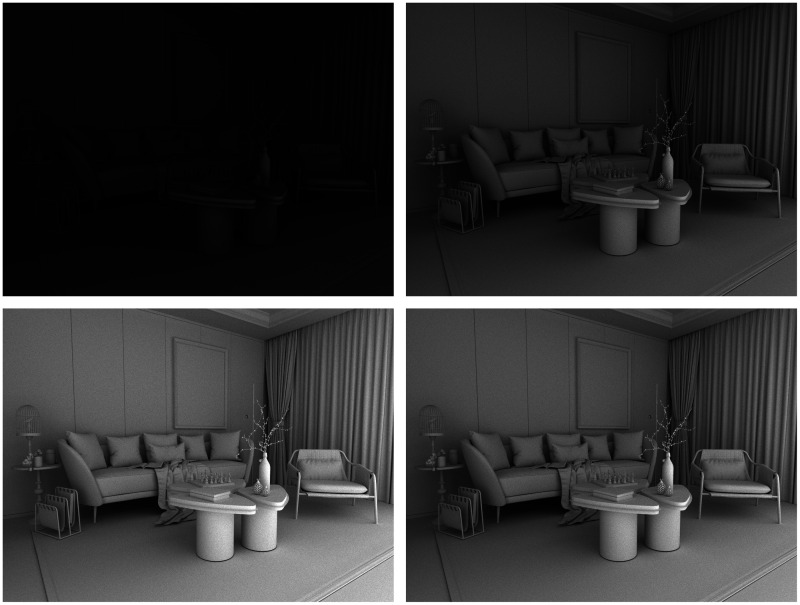
Four images of a white room with varying degrees of interreflection. Moving clockwise from the upper left, the images were rendered 1, 5, 10 and 15 indirect bounces, respectively.

Another convenient way to demonstrate the perceptual importance of surface interreflections is to examine scenes with no direct reflections at all. This is actually a fairly common occurrence. Most people find it unpleasant to look directly at luminous sources so we typically hide them behind baffles or translucent diffusers. Consider the four images in Figure 9. They all depict a living room in which all the surfaces reflect 85% of the incident light. The scenes are all illuminated by a single spherical light source located behind a baffle so there is no direct illumination on any of the visible surfaces. These images were created with a renderer that allows one to manipulate the number of indirect bounces, and that is the only thing that differs between them. Moving clockwise from the upper left, the images show the rendering results for 1, 5, 10, and 15 indirect bounces, respectively.

Gilchrist and Jacobsen were way ahead of their time in recognizing the perceptual importance of indirect reflections, and the impact of their research may have suffered accordingly. A very similar experiment on color constancy was performed 15 years later by Bloj et al. (1999), but that one was published in *Nature*. The psychophysical study of lighting effects including shadows and indirect reflections began to take off around that time, with Dan Kersten and his colleagues at the University of Minnesota leading the way (e.g., see Kersten et al., 1996; Madison et al., 2001; Mamassian et al., 1998). They were one of the few groups in the world at that time with the resources and expertise to conduct that research.

**Figure 10.**
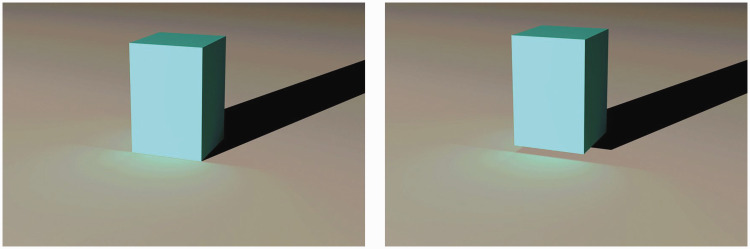
Effects of shadows and indirect illumination on the perception of surface contact.

The work of Madison et al. (2001) on the perception of surface contact is particularly relevant in this regard because it investigated the effects of both cast shadows and indirect illumination. Gibson (1950) had argued that the projected optical adjacency of an object and the ground is the relevant source of information for the perception of surface contact, which can sometimes lead to perceptual illusions when an object is actually not in contact with the ground. Madison et al. (2001) showed that optical adjacency can be overridden by other sources of information. Figure 10 shows a recreation of two of their stimuli that depict a rectangular box and a planar ground surface. The box on the left appears to be resting on the ground, whereas the one on the right appears to be hovering above the ground. The relevant information that is responsible for this difference is the placement of the shadow or indirect illumination relative to the base of the object. When they are properly attached, the box appears on the ground, but when they are separated, the box appears floating above the ground. This effect also works if the shadow is presented without indirect illumination, or if the indirect illumination is presented without the shadow. However, if both of them are removed, the box always appears in contact with the ground.

## The Visual Perception of the Light Field

**Figure 11.**
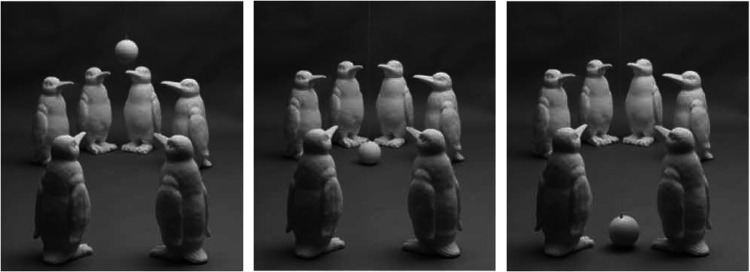
Some stimulus probe locations for Koenderink et al. (2007).

One of the things that was emphasized by both Gibson and Gershun is that light in the environment has a complex structure that fills the volume of space. This raises an interesting question about what, if anything, observers know about that structure. The first experiment to address that issue was performed by Koenderink et al. (2007). The stimuli in this experiment consisted of a circular array of penguins with three possible patterns of illumination: (a) a distant point light similar to a bright sunny day, (b) a diffuse overhead light similar to an overcast day, and (c) a spherical area light in the center of the configuration similar to a camp fire at night. To measure observers’ knowledge about the pattern of radiant flux within these scenes, a small spherical probe was placed at a series of different locations, and observers were required to adjust the pattern of shading on the probe at each location so that it appeared to be consistent with the shading in the rest of the scene. This was achieved using a four-dimensional adjustment in which they could manipulate the intensity, diffuseness, and direction of illumination. Figure 11 shows three of the possible probe placements for the cloudy day condition with the correct pattern of shading.

**Figure 12.**
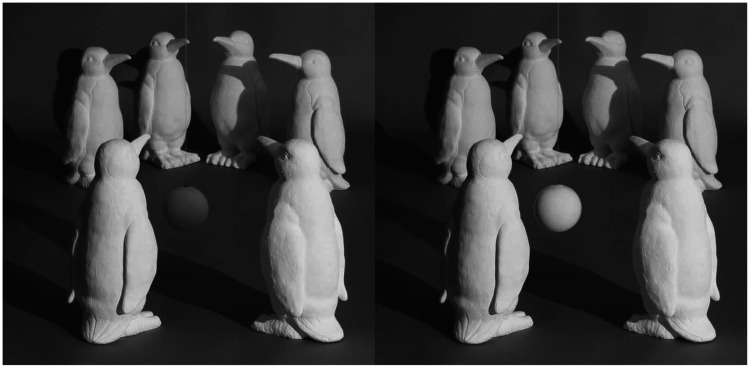
Errors with volumetric shadows. The left panel shows the correct setting and the right panel shows the average observer setting.

Although four-dimensional adjustment spaces are generally ill-advised in psychophysical research, the observers found this one to be quite natural, and their responses on the whole were remarkably accurate. However, they did produce one systematic pattern of errors in the sunny day condition, which is demonstrated in Figure 12. The left panel of this figure shows a probe in that condition with the correct pattern of shading. Note that the probe is relatively dark because it is within the shadow of the front-right penguin. The right panel shows the same probe with the average setting for the actual observers. Note in this case that the probe is much brighter than it should be because observers do not take into account the volumetric region for which the direct light is occluded.

**Figure 13.**
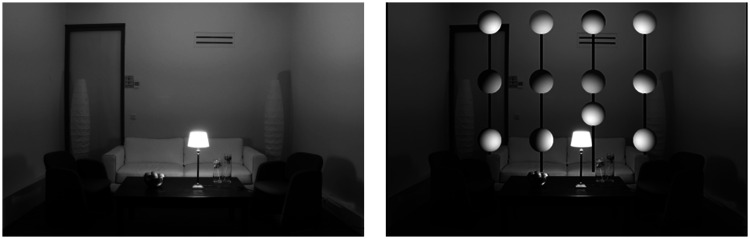
A 3D scene used by Kartashova et al. (2016; left panel) and a sample of the probe regions (right panel).

Following that original experiment by Koenderink et al. (2007), this light probe procedure has been further developed by Sylvia Pont and her colleagues at Delft Technical University. For example, in one recent study by Kartashova et al. (2016), both physical and psychophysical measures of the light field were obtained. The left panel of Figure 13 shows one of the scenes employed in that research, and the right panel shows many of the probe locations for the psychophysical measurements. Physical measures of the luminous flux were obtained at the same locations using a small box that contained several photometers pointing in different directions.

**Figure 14.**
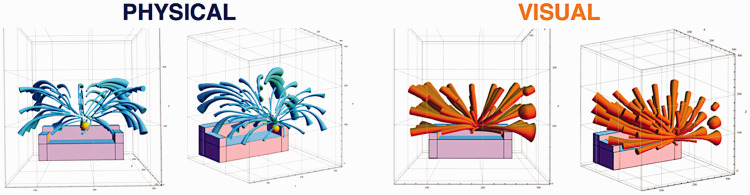
The light fields obtained by Kartashova et al. (2016) for physical measures (Left) and perceptual measures (Right). The depicted tubes point in the maximum direction of luminous flux at each location, and the intensity of the light is inversely proportional to the width of the tube.

The left panel of Figure 14 shows a summary of the physical measures. The method of visualization depicts a pattern of curved tubes as was first suggested by Gershun (1939). The direction of a tube at any given location represents the primary direction of luminous flux at that location, and the widths of the tubes are inversely proportional to the light intensity. Note in particular that there is a considerable amount of curl in the light field due to indirect illumination off the walls and ceiling. The right panel of Figure 14 shows the same representation for the psychophysical measures. Note in that case that the curl is almost entirely absent.

When considered in combination, these results indicate that observers have some knowledge of the light field, but that they do not take into account the higher order effects of volumetric shadows and indirect illumination. How might we reconcile these findings with other results described earlier that these higher order effects of the light field can have a significant influence on the perception of surface layout and on color and lightness constancy? I suspect that our perceptual knowledge about shadows and indirect illumination is quite limited. We typically think of shadows as being projected on the ground as opposed to filling a volume of space, but the latter is a more accurate characterization. Similarly, we can know about the presence of surface interreflections because of the softening of shadows they produce, but we are apparently clueless about how this affects the directions of luminous flux at any given location.

## Living in the Material World

In his discussion of reflective scattering, Gibson noted that the scattering of light on matte materials is distributed over a broad range of directions, whereas the scattering on glossy materials is more tightly clustered within a small range of directions (see Figure 3). These observations are quite similar to the reflectance models that were used for shaded computer graphics in the 1970s and 1980s. Although these models could be parameterized in a variety of ways, they almost always included a diffuse component (with high scattering) and a specular component (with low scattering) that could be combined additively in different proportions.

All of that began to change in the 1990s. In their never ending quest for photorealism, researchers in computer graphics (and other related fields) started to expand the repertoire of materials they could successfully simulate. I first became aware of this research while visiting Jan Koenderink and Ans Van Doorn at the University of Utrecht. Together with Sylvia Pont, they were attacking this problem on several different fronts: They made physical measurements of the bidirectional reflectance distribution functions (BRDF) for a wide range of materials. They built large scale models of the microscopic surface structure to explain how these BRDFs arise. They attempted to write mathematical equations that could accurately fit their data, and they also tried to identify the relevant visual information by which these materials might be perceptually identified. As a result of this extensive investigation, Pont and te Pas (2006) proposed four theoretical BRDFs that represent generic types of surface materials that occur in the natural environment. These include diffuse (Lambertian) reflection on matte surfaces, specular (forward) reflection on glossy surfaces, backscattering on rough surfaces, and asperity scattering on surfaces with fine hairs such as peach, skin, or velvet.

Similar research was performed in many other laboratories around the world, resulting in several publically available archives of BRDF data. This growing body of knowledge was eventually incorporated into commercial renderers. Many of these packages now contain powerful material editors that allow users to create simulated materials with an astonishing degree of flexibility, including effects such as subsurface scattering on translucent materials (Jensen et al., 2001), or thin film interference for coated materials.

**Figure 15.**
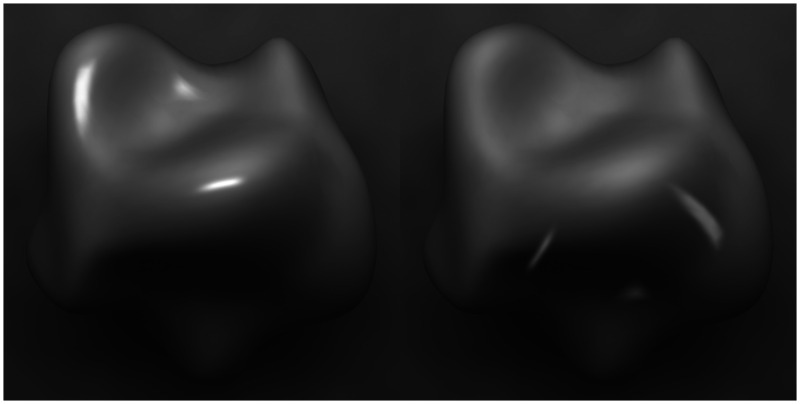
In order for regions of high contrast in an image to be perceived as specular highlights, they must be aligned appropriately with directions of minimum curvature on a surface.

One of the first psychophysical demonstrations involving surface material properties (other than color or lightness) was presented by Beck and Prazdny (1981). They superimposed artificial highlights on a digitized photograph of a vase. When these highlights were oriented along directions of minimum curvature on the object, it was judged to be significantly more glossy than when the highlights were oriented in the direction of maximum curvature. The two images shown in Figure 15 were created by Todd et al. (2004) in an effort to replicate their original demonstration. Note in the left panel how the highlights are all oriented along directions of minimum curvature on the surface, which is how they behave in most natural viewing contexts. If the highlights are rotated relative to the diffuse shading so that they no longer conform to that constraint, as shown in the right panel, then they appear as stray light or patches of white paint on a surface. A more systematic investigation of this phenomenon was performed later by Anderson and Kim (2009). They correctly recognized that it provides an important counterexample to a hypothesis proposed by Motoyoshi et al. (2007) that glossy surfaces can be identified by the statistical distribution of pixel intensities in an image, without considering how highlights are arranged with respect to the surface geometry.

**Figure 16.**
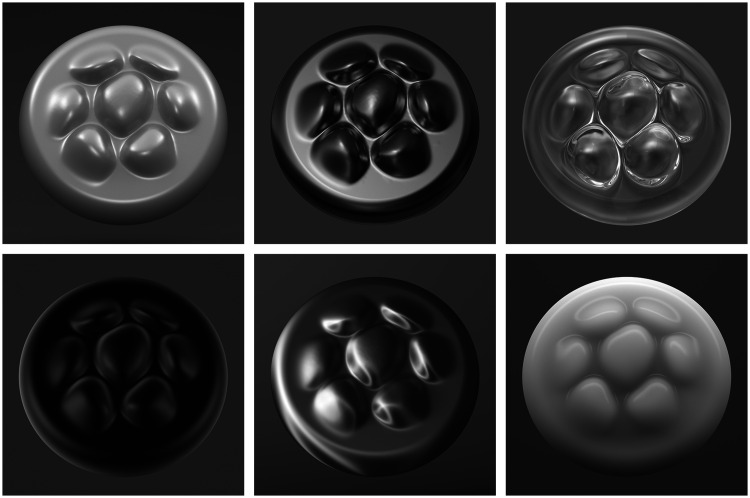
Images of a single object with different patterns of illumination and different surface materials. the top row from left to right depicts shiny white plastic, metal and glass. The bottom row depicts black velvet, satin and wax.

During the past two decades, there have been many psychophysical investigations of the perceptual distinction between matte and glossy materials (e.g., Doerschne et al., 2007, 2010; Kim et al., 2011, 2012; Marlow & Anderson, 2013), and this research has produced some surprising results. Although observers’ judgments are indeed influenced by the pattern of surface scattering, as suggested by Gibson, they are also influenced by other factors such as the 3D shape of an object and its pattern of illumination.

Of course our perception of surface materials is much richer than the limited distinction between matte and glossy, and the psychophysical research to explore that richness is expanding at a rapid rate. Some noteworthy examples of this include the perception of translucency due to subsurface scattering within solid 3D objects (Fleming & Bülthoff, 2005; Marlow et al., 2017; Xiao et al., 2014), the perceptual distinctions among different types of shiny materials such as metal or plastic (Norman et al., 2020; Todd & Norman, 2018), the perception of velvet (F. Zhang et al., 2015), and the perception of glass (Fleming et al., 2011; Todd & Norman, 2019).

This expanded ability to manipulate surface material properties opens up another avenue of research that has been largely neglected in the literature. It is important to keep in mind that almost all computational analyses of 3D shape from shading are based on strong assumptions that (a) visible surfaces scatter light uniformly in all directions and (b) all points on a surface are homogeneously illuminated from the same direction. These traditional analyses typically fail catastrophically whenever those underlying assumptions are violated (R. Zhang & Tsai, 1999). In other words, they do not exhibit shape constancy over variations in illumination or surface materials.

This raises an important question of whether the visual perception of 3D shape from shading is more robust to these types of changes. Consider the array of images depicted in Figure 16, which all depict a circular puck with a pattern of bumps on its top surface. All of these objects are illuminated differently and they are all composed of different materials, which include shiny white plastic, metal, glass, black velvet, satin, and wax. The initial impression conveyed by these images is that the depicted objects all have the same shape, although there are some subtle differences that become evident upon closer inspection. For example, the bumps on the wax object appear slightly flatter than the others, and the ones on the glass object appear slightly more pointed. This demonstration suggests that our perception of 3D shape has a high degree of constancy over changes in illumination or material properties and that is consistent with recent empirical findings by Egan and Todd (2015) and Todd et al. (2014). However, the literature is not consistent on this point. Other researchers have done similar experiments and found much larger violations of shape constancy (e.g., Khang et al., 2007; Pont & te Pas, 2006).

It is especially interesting to note in this context that there is a new computational analysis of 3D shape from shading developed by Kunsberg and Zucker (2018) that relies on much weaker assumptions than previous approaches. One important insight that underlies this model is that different regions of a shaded image are not equally informative. In general, the curves that connect points of equal intensity in an image (called isophotes) undergo large variations over changes in the pattern of illumination, but they remain much more stable in regions of high curvature. By focusing specifically on those regions, Kunsberg and Zucker’s model is able to achieve a higher degree of illumination invariance than is possible with other approaches.

This could provide a possible explanation of the discrepant findings on shape constancy described earlier. Note in Figure 16 that the depicted object has many regions of high curvature and that is also the case for the objects used by Egan and Todd (2015) and Todd et al. (2014). In contrast, the objects used by Khang et al. (2007) and Pont and te Pas (2006) were limited to ellipsoidal surfaces, which may not be the ideal surface geometry for the perception of 3D shape from shading. Additional research is clearly necessary in order to test that hypothesis.

## Summary and Conclusions

This special issue of *i-Perception* was inspired by a 2019 symposium to celebrate the 40th anniversary of Gibson’s final book at the European Conference on Visual Perception in Leuven. To select a specific topic for my talk at that symposium, I took the opportunity to reread all three of his books for the first time in at least three decades. Most of what I encountered was quite consistent with my memories from earlier readings, but when I got to the chapter on the ambient optic array in the 1966 book, I was absolutely blown away. When I reread that chapter with the benefit of hindsight, I could immediately see connections I had never noticed before to problems I had been studying throughout my career. I later described this to one of my colleagues, Flip Phillips, and he reported having exactly the same experience when he recently reread Gibson. That is when I decided that I would focus my talk on the ambient optic array and that I would write an article on the same topic.

Gibson had a profound influence on the field of visual perception, and many of the concepts he introduced are still being investigated today. For example, no one would write a paper on optical flow without acknowledging Gibson’s seminal contributions in that area. Nor would one write a paper on the perception of slant from optical texture without citing Gibson. It is quite curious, therefore, that his name is conspicuously absent from the literature on the perceptual analysis of image shading. His discussion of shading gradients in chiaroscuro is no less clear than his discussion of texture or flow gradients, and he correctly identified almost all of the basic optical principles that are necessary to simulate patterns of shading or to investigate their perceptual interpretation.

It is interesting to speculate about why his analysis failed to inspire a focused program of research on this topic. I believe that there are two primary reasons for this. First, the phenomenon of chiaroscuro is much more complex than other sources of optical information he identified, such as gradients of texture or optical flow. Those can be successfully analyzed using only projective geometry, but in order to understand patterns of shading it is necessary to explicitly model how light interacts with surfaces. The field of visual perception was just not ready for that level of complexity in the 1960s. Second, even if others more clever than I had been able to appreciate the significance of Gibson’s ideas about the behavior of light, they would likely have been unable to develop a coherent research program on how chiaroscuro influences visual perception. To achieve that, they would have needed to systematically manipulate patterns of shading in some way to create an appropriate set of stimuli. The tools to do that were simply unavailable back then, and when they did become available 15 years later, the precise details of his insightful discussion about the behavior of light had been overshadowed by other concepts he had stressed more heavily in his subsequent writings.

More than anything else, it was the invention of raster computer graphics, and the enormous effort to achieve photorealism with that technology that inspired young researchers like me in the 1980s to pursue a program of research on the perceptual analysis of image shading. Moreover, this is not a topic where the earliest researchers could lay claim to all of the important discoveries. As computers become faster and faster, the field of computer graphics continues to develop accurate simulations of more and more complex visual phenomena, such as the dynamics of cloth, hair, and fluid flows. This in turn provides additional motivation for newer generations of young researchers to investigate problems that could not have been studied previously (e.g., Kawabe et al., 2015; Schmid & Doerschner, 2018; Van Assen et al., 2018).

I suspect that if Gibson were around today, he would be delighted by how the field has evolved toward what he was advocating 50 years ago. His dream of a science of ecological optics formed from an amalgamation of ideas from many different fields has largely come to pass. It is difficult to know to what extent, if any, his writings may have influenced the early development of computer graphics. It is important to keep in mind that the earliest research on global illumination was performed at Cornell University, and at least some of the members of that team took courses from Gibson. Although he was brilliant in his conceptual description of the behavior of light, he could not provide some of the necessary tools for computer simulations that were available in illumination engineering, like physical measurements of reflected light and equations to accurately fit those measurements. Nevertheless, even if it could be shown that his impact on these developments may have been minimal, we can still look back and admire the scope of his insights. He was remarkably prescient in his analysis of chiaroscuro by precisely anticipating how research in computer graphics would unfold many years later. That is what blew me away when I reread his work with the benefit of hindsight, and I believe it provides a compelling testament to his genius.
